# Transcriptome profiling analysis reveals the role of silique in controlling seed oil content in *Brassica napus*

**DOI:** 10.1371/journal.pone.0179027

**Published:** 2017-06-08

**Authors:** Ke-Lin Huang, Mei-Li Zhang, Guang-Jing Ma, Huan Wu, Xiao-Ming Wu, Feng Ren, Xue-Bao Li

**Affiliations:** 1 Hubei Key Laboratory of Genetic Regulation and Integrative Biology, School of Life Sciences, Central China Normal University, Wuhan, China; 2 Oil Crops Research Institute, Chinese Academy of Agricultural Sciences, Wuhan, China; Huazhong University of Science and Technology, CHINA

## Abstract

Seed oil content is an important agronomic trait in oilseed rape. However, the molecular mechanism of oil accumulation in rapeseeds is unclear so far. In this report, RNA sequencing technique (RNA-Seq) was performed to explore differentially expressed genes in siliques of two *Brassica napus* lines (HFA and LFA which contain high and low oil contents in seeds, respectively) at 15 and 25 days after pollination (DAP). The RNA-Seq results showed that 65746 and 66033 genes were detected in siliques of low oil content line at 15 and 25 DAP, and 65236 and 65211 genes were detected in siliques of high oil content line at 15 and 25 DAP, respectively. By comparative analysis, the differentially expressed genes (DEGs) were identified in siliques of these lines. The DEGs were involved in multiple pathways, including metabolic pathways, biosynthesis of secondary metabolic, photosynthesis, pyruvate metabolism, fatty metabolism, glycophospholipid metabolism, and DNA binding. Also, DEGs were related to photosynthesis, starch and sugar metabolism, pyruvate metabolism, and lipid metabolism at different developmental stage, resulting in the differential oil accumulation in seeds. Furthermore, RNA-Seq and qRT-PCR data revealed that some transcription factors positively regulate seed oil content. Thus, our data provide the valuable information for further exploring the molecular mechanism of lipid biosynthesis and oil accumulation in *B*. *nupus*.

## Introduction

*Brassica napus* (rape crop, AACC, 2n = 38) is one of the primary sources of oil which is mainly in the form of triacylglycerols (TAGs) and widely grows in China, Canada, Europe, Australia and South America [1]. Not only does rapeseed oil serve as vegetable oil for human nutrition and occupy a pivotal position on oil supply in China, but also there has been increased interest in these oils as a source for the production of lubricants, inks, paints, and biofuel [2–4]. With the increasing demand for rapeseed oil in both food and non-food application, the economic and scientific interest with regard to the regulation and dynamics of seed oil accumulation in *B*. *nupus* is growing [5, 6].

Under the control of three genetic effects (embryonic, cytoplasmic and maternal), oil content of rapeseeds is a complicated quantitative trait, and lipids synthesis in *B*. *napus* is also dependent on interaction between the genotype and environment [7, 8]. De novo fatty acid (FA) synthesis and triacylglycerol assembly are tightly linked to silique wall photosynthesis and carbohydrate metabolism, especially the starch metabolism and pyruvate metabolism, which provide carbon source for FA synthesis. Sucrose is mainly produced in the pod wall during the seed-filling stage in rape plants. The above biology processes are controlled by maternal genotype and environment. Sucrose is then transported into developing seeds and converted into fructose and UDP-glucose (hexose). Hexose is further converted into acetyl-CoA which is the precursor of de novo fatty acid synthesis in the cytosol and the plastid of the embryo cells [9–12]. Hence, a comprehensive consideration of embryonic, cytoplasmic, and maternal effects is important for uncovering the molecular mechanism of oil accumulation.

Over last decade, the growth in knowledge regarding lipid biosynthetic pathways and genes involved in lipid biosynthesis in embryos has accelerated our understanding of seed oil biosynthesis and bioaccumulation, especially in the model plant *Arabidopsis thaliana* [6]. Identification of several hundred genes involved in lipid biosynthesis has been facilitated by extensive annotation of the *Arabidopsis* genome [13,14]. The microarray analysis and conventional expressed sequence tag (EST) sequencing have revealed their transcription patterns and the potential transcription factors involved in storage lipid metabolism during seed maturation. The intricate transcription regulatory system that controls *Arabidopsis* seed development has been determined[15–17]. B3 domain superfamily of plant-specific DNA-binding proteins AtABI3[18, 19], AtLEC1[20], AtLEC2[19, 21], AtbZIP53[22] and AtFUS3 [19, 23] are master regulators of the maturation process and reserve accumulation in *Arabidopsis*. AtWRI1, a transcription factor of the APETALA2-ethylene responsive element-binding protein (AP2-EREBP) family that specifies the regulatory action of *AtLEC2* and possibly *AtLEC1* toward the fatty acid biosynthetic network, has been reported in the control of genes encoding enzymes for plastidial glycolysis and fatty acid biosynthesis [21, 24–27]. A set of three closely related AtVAL/AtHIS B3 domain factors, PKL and ASIL1 shuts down the maturation program before germination [28–34]. The PII/AtGLB1 protein interacts with BCCP subunits of heteromeric HtACCase in a 2-oxoglutarate-dependent manner and controls ACCase activity by reducing the Vmax of the enzyme [35]. All of these researches have centered on oil synthesis in developing *Arabidopsis* embryos. However, only few studies have attempted to understand the interplay between TFs and their target genes in rapeseed plant species, such as *B*. *napus*.

Genetic-based studies revealed that maternal effects play a critical role in controlling seed oil content in *Arabidopsis* [36], *B*. *napus* [37], soybean [38], and flax [39]. Moreover, additional studies concerning the underlying mechanisms of these effects are essential to fully understand oil synthesis regulation. However, we still know relatively little about how lipid synthesis is regulated in *B*. *napus* and how oil accumulates in and outside of rapeseeds. Especially the molecular mechanism of combined effect of embryonic, cytoplasmic and maternal is unclear so far. The lack of understanding hinders the development of future plant breeding [37, 40–43]. In this study, RNA sequencing technique (RNA-Seq) was performed to explore differentially expressed genes in siliques of two lines of *B*. *napus* with contrasting oil content at 15 and 25 days after pollination (DAP). We focused on the comprehensive embryonic, cytoplasmic and maternal effects on silique development and rapeseed oil content. The results provide theoretical basis for the future molecular breeding in *B*. *napus*.

## Materials and methods

### Plant materials

Two lines HFA (high fatty acids, approximately 43.87% oil content in seeds) and LFA (low fatty acids, approximately 31.74% oil content in seeds) were selected from the filial generations of *Brassica napus* cultivars G166 and ZheShuang6 (both are typical German semi-winter rape cultivars) for this study. The HFA and LFA materials were provided by Oil Crops Research Institute, Chinese Academy of Agricultural Sciences, and the oilseed rape plants grew under normal conditions in experimental field in campus of Central China Normal University, Wuhan, China.

### RNA isolation and quantitative RT-PCR analysis

The siliques were collected at different stages, which is based on the results of emasculation, bagging and artificial pollination when the flower was ready to bloom. The siliques were gently grinded in liquid nitrogen, and then the seeds were picked out in liquid nitrogen using tweezers for RNA extraction. Total RNA was extracted from 10, 15, 20, 25, 30, 35, 40, 45 and 50 DAP (days after pollination) seeds and silique walls, and purified using RNeasy Mini kit (Qiagen, German).

The expression of genes was analyzed by real-time quantitative RT-PCR using the fluorescent intercalating dye SYBR Green in a detection system (MJ Research, Opticon 2). The *BnActin2* (*BnACT2*) gene was used as standard control in the quantitative RT-PCR reactions. Two-step RT-PCR procedure was performed using a method described previously [44]. The genes detected by RT-PCR were analyzed by basic local alignment search tool (http://www.genoscope.cns.fr/blat-server/cgi-bin/colza/webBlat), and multiple sequences alignment of homologous genes was completed by Clustal W. Then, the gene-specific primers were designed based on multiple sequences alignment (S1 Table).

### Library construction and sequencing

A total of 1 μg RNA per sample from 15 and 25 DAP siliques was used for the RNA sample preparations. Sequencing libraries were generated using NEBNext^®^ Ultra^™^ RNA Library Prep Kit for Illumina^®^ (NEB, USA) following manufacturer’s instruction. Briefly, mRNA was purified from total RNA using poly-T oligo-attached magnetic beads. Fragmentation was carried out using divalent cations under elevated temperature in First Strand Synthesis Reaction Buffer. First strand cDNA was synthesized using random hexamer primer and M-MuLV Reverse Transcriptase (RNase H-). Second strand cDNA synthesis was subsequently performed using DNA polymerase I and RNase H. Remaining overhangs were converted into blunt ends via exonuclease/polymerase activities. After adenylation of 3’ ends of DNA fragments, adaptor was ligated for hybridization. In order to select cDNA fragments in length, the library fragments were purified with AMPure XP system (Beckman Coulter, Beverly, USA). Then USER Enzyme (NEB, USA) was used with size-selected and adaptor-ligated cDNA at 37°C for 15 min followed by 5 min at 95°C before PCR. PCR was performed with Phusion High-Fidelity DNA polymerase, Universal PCR primers and Index (X) Primer. The PCR products were purified with AMPure XP system and library quality was assessed on the Agilent Bioanalyzer 2100 system. Finally, the four libraries were sequenced using an Illumina HiSeq2500^™^ platform with a read length of 125 (PE125, paired-end).

### Quality control and reads mapping to the reference genome

Raw reads produced from sequencing machines contain dirty reads which contain adapters, unknown or low quality bases. These data will negatively affect following bioinformatics analysis. Therefore, dirty raw reads are removed: 1. Remove reads with adaptors; 2. Remove reads with unknown nucleotides larger than 5%; 3. Remove low quality reads (The rate of reads which quality value ≤ 10 is more than 50%).

The *Brassica napus* genome databases (http://www.genoscope.cns.fr/brassicanapus/) were used as a reference, and gene model annotation files were downloaded from genome website directly. Clean reads were aligned to the reference genome using HISAT. HISAT (http://www.nature.com/nmeth/journal/v12/n4/full/nmeth.3317.html) was published in Nature Method in 2015 with a better mapping accuracy than other mapping tools.

### Quantification of gene expression level and differential expression analysis

HTSeq v0.6.1 was used to count the reads numbers mapped to each gene. And then the calculation of gene expression uses RPKM method (Reads Per kb per Million reads). The formula is shown below:

$$RPKM=\frac{10^{6}C}{NL/10^{3}}$$

Differential expression analysis was performed using the DEGSeq, qvalue (or FDR) <0.001 & |log2(foldchange)|>1 was set as the threshold for significantly differential expression.

### GO enrichment analysis of DEGS

Gene Ontology (GO) enrichment analysis of differentially expressed genes was implemented by the GOseq, in which gene length bias was corrected. GO functional analysis provides GO functional classification annotation for DEGs as well as GO functional enrichment analysis for DEGs. GO was generated using Gene Ontology database (http://www.geneontology.org/).

### KEGG pathway enrichment analysis

Different genes usually cooperate with each other to exercise their biological functions. Pathway-based analysis helps to further understand genes biological functions. KEGG is the major public pathway-related database (http://www.genome.jp/kegg/). KOBAS software was used to test the statistical enrichment of differential expression genes in KEGG pathways.

## Results

### Mapping of RNA-Seq data and evaluation of differentially expressed genes

To investigate whether siliques play a role in regulation of seed oil content, we performed RNA sequencing (RNA-Seq) analysis of *Brassica napus* siliques. We observed the seed morphology and detected the expression levels of three marker genes *BnBCCP* (biotin carboxyl carrier protein), *BnFAD* (fatty acid desaturase) and *BnWRI1* in seeds of two *B*. *napus* lines (HFA and LFA) at different developmental stages. As shown in S1B Fig, the seed morphology of HFA and LFA are similar at different developmental stages. The seed had generally attained their final size at ~20 DAP and the seed remained green during 20–35 DAP which are stage of reserve accumulation at the seed maturation stage [45]. In addition, *BnBCCP*, *BnFAD* and *BnWRI1* were expressed at the highest levels in developing seeds at 25 DAP, suggesting that 25 DAP may be the key stage for seed oil accumulation (S1A Fig). Four cDNA libraries were constructed using RNA isolated from siliques of HFA and LFA at 15 and 25 DAP (namely LFA15, LFA25, HFA15 and HFA25), respectively, and large-scale sequenced. Approximately, 49.78–50.86 million high cleaned raw quality reads were generated in each individual sample after the quality control. Among them, 74%– 80% clean reads were uniquely mapped in *B*. *napus* genome (http://www.genoscope.cns.fr/brassicanapus/) by HISAT mapping tool (see Methods). The number of genes with a FPKM (fragments per kilobase of exon per million fragments mapped) value > 0 for each treatment was 65746 (LFA15), 66033 (LFA25), 65236 (HFA15) and 65211 (HFA25), respectively (Table 1). It is worth noting that we detected 944 novel transcripts, of which 698 transcripts are coding transcripts and 246 transcripts are non-coding transcripts (S1 Dataset).

**Table 1:** Reads number based on the RNA-Seq data and distribution of all genes detected in libraries of LFA15, LFA25, HFA15 and HFA25.

	High quality reads	Unique mapped reads	Read mapped Gene	Detected Gene Number
LFA15	49976820	37481782(75.29%)	30920658(61.87%)	65746
LFA25	49783214	37192749(74.42%)	31582471(63.44%)	66033
HFA15	50868374	40832043(80.27%)	35017789(68.84%)	65236
HFA25	50844144	40731244(80.11%)	34843492(68.53%)	65211

**Fig 1:**
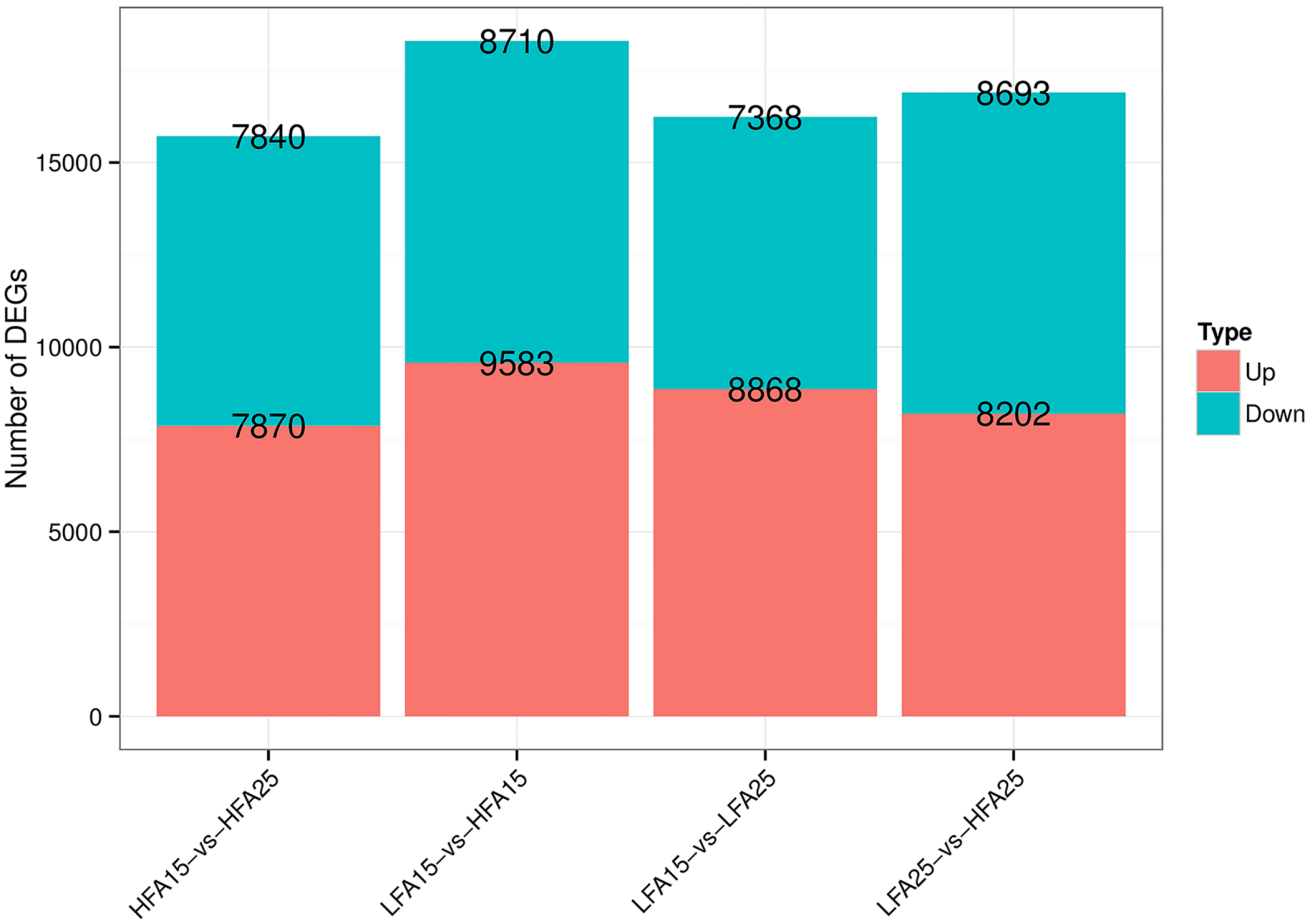
Total numbers of the differentially expressed genes (DEGs) between LFA15, LFA25, HFA15 and HFA25 of *B*. *napus*.

To explore an overview of interesting genes, we used the DEGSeq to find the differentially expressed genes (DEGs) between these four samples. The DEGs were defined as the fold change of FPKM expression values and were at least 2 in either direction when t q-value or FDR < 0.001 and the absolute value of log2 (fold change) > 1. A large number of DEGs were identified among these samples. The numbers of up-regulated and down-regulated genes of 15 DAP and 25 DAP in the two different fatty acid contained materials’ siliques were presented in Fig 1. To validate whether the RNA-Seq data are reliable, we randomly selected some DEGs to further analyze their expressions in the four materials by RT-PCR. As shown in S2 Fig, the expression patterns of these genes were consistent with the RNA-Seq data.

### Gene ontology (GO) enrichment analysis of differentially expressed genes (DEGs)

**Fig 2:**
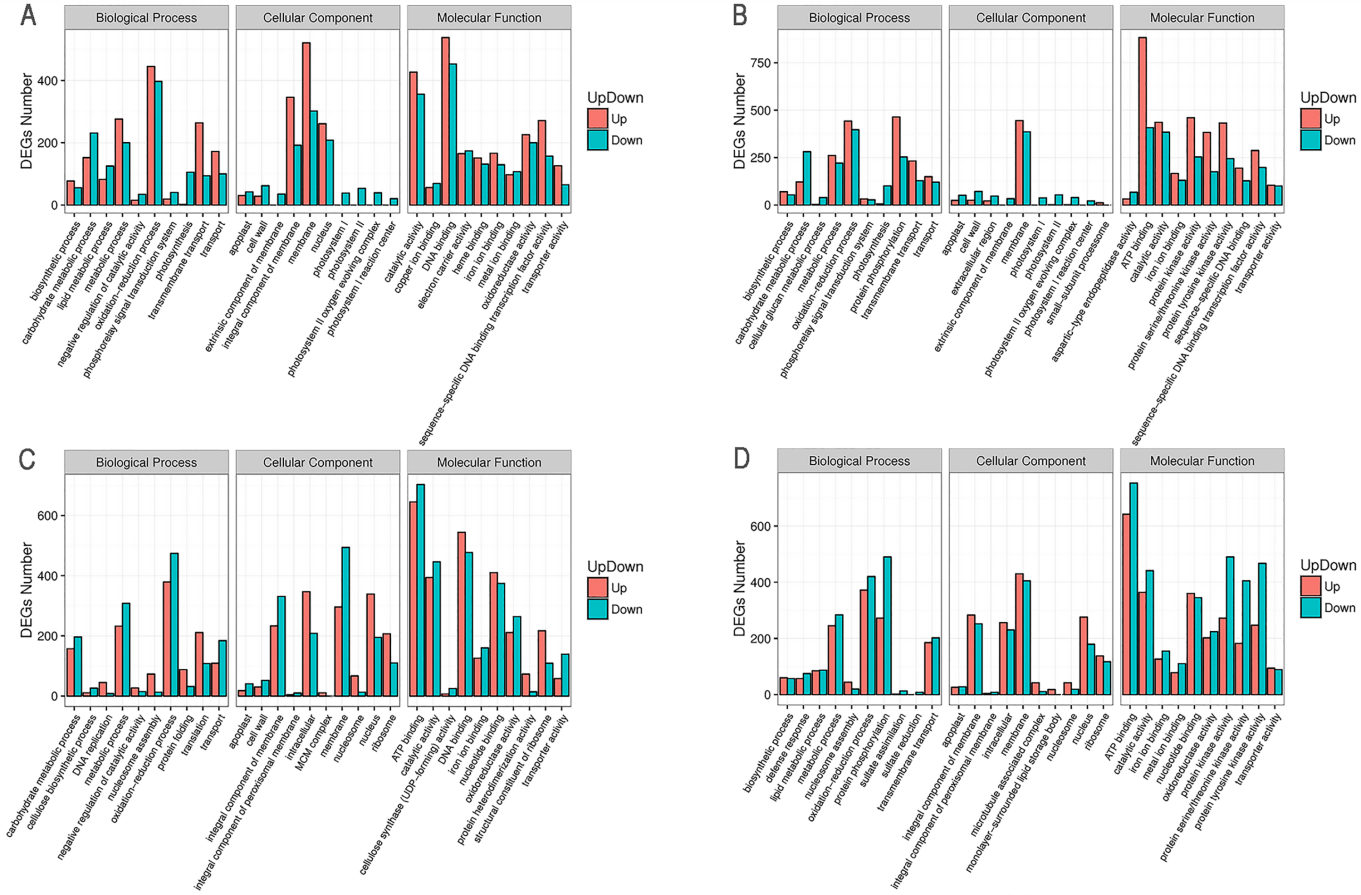
Gene Ontology (GO) classification of the differentially expressed genes (DEGs) among LFA15, LFA25, HFA15 and HFA25 of *B*. *napus*. (A) The number of a specific category of the upregulated and downregulated genes in HFA15 vs HFA25. (B) The number of a specific category of the upregulated and downregulated genes in LFA15 vs LFA25. (C) The number of a specific category of the upregulated and downregulated genes in LFA15 vs HFA15. (D) The number of a specific category of the upregulated and downregulated genes in LFA25 vs HFA25. Genes were annotated in three categories: biological process, cellular component and molecular function. Y-axis represents the number of a specific category of the upregulated and downregulated genes.

To obtain ontology of defined terms concerning gene product properties, we performed the gene ontology (GO) analysis. As shown in Fig 2 and S2 Dataset, the GO category could divide all genes into three major groups: cellular component, molecular function and biological process. Using GO database (http://www.geneontology.org), the genes were classified into corresponding annotated functional subcategories.

To know the significant terms of the DEGs, the GO enrichment analysis of up-regulated and down-regulated genes of HFA15 vs HFA25, LFA15 vs LFA25, LFA15 vs HFA15, and LFA25 vs HFA25 was completed against the background of GO term distribution of all expression genes, and the most enriched thirty GO terms are shown in Fig 2 and S2 Dataset. The most enrichment five GO subcategories of LFA15 vs HFA15 are ATP binding (GO:0005524), DNA binding (GO:0051090), oxidation-reduction process (GO:0055114), catalytic activity (GO:0043086) and nucleotide binding (GO:0000166). Similarly, for LFA25 vs HFA25, the most enrichment five GO subcategories are protein binding (GO:0005515), ATP binding (GO:0005524), DNA binding (GO:0051090), catalytic activity (GO:0055114) and oxidation-reduction process (GO:0055114). On the other hand, the most enrichment five GO subcategories of LFA15 vs LFA25 are ATP binding (GO:0005524), DNA binding (GO:0051090), oxidation-reduction process (GO:0055114), membrane (GO:0006855) and catalytic activity (GO:0043086), while the most enrichment five GO subcategories of HFA15 vs HFA25 are DNA binding (GO:0051090), oxidation-reduction process (GO:0055114), membrane (GO:0006855), catalytic activity (GO:0043086) and integral component of membrane (GO:0016021) which may play an important role in pod development and seed oil accumulation.

Apparently, the genes related DNA binding, catalytic activity and oxidation-reduction process are those with the most significantly differential expression in all GO subcategories among the four samples. Meanwhile, some differences were also found when we compared the up-regulated and down-regulated subcategories. For example, one of the most enrichment five GO subcategories of LFA25 vs HFA25 DEGs is protein binding, which is different from HFA15 vs HFA25, LFA15 vs LFA25, and LFA15 vs HFA15. The function subcategories represent the different gene expression patterns among these four samples. These results may offer us some useful information to understand the molecular base of pod development and seed lipid synthesis in *B*. *napus*.

### KEGG pathway enrichment analysis of DEGs

**Fig 3:**
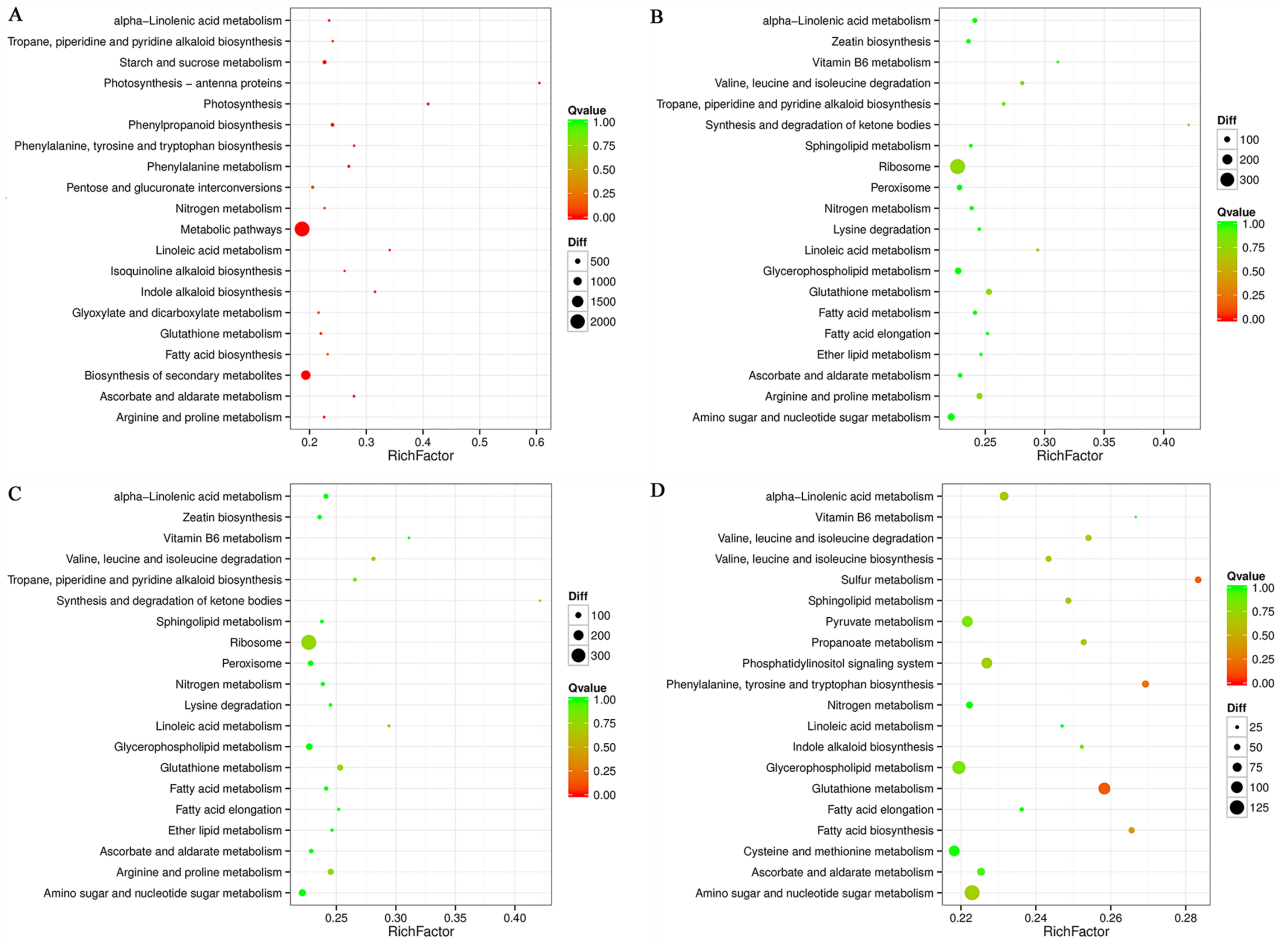
KEGG pathway categories of differentially expressed genes (DEGs) among LFA15, LFA25, HFA15, and HFA25 of *B*. *napus*. (A) KEGG pathway categories of DEGs in HFA15 vs HFA25. (B) KEGG pathway categories of DEGs in LFA15 vs LFA25. (C) KEGG pathway categories of DEGs in LFA15 vs HFA15. (D) KEGG pathway categories of DEGs in LFA25 vs HFA25. The X-axis (Rich factor) represents the proportion of DEG accounted for all genes of a specific KO term. The size of the point represents the number of related DEGs. The Q value is the calibration of p value.

Different genes usually cooperate with each other to exercise their biological functions. Analyzing the pathway annotations help to further interpret the biological functions of the differently expressed genes. KEGG (Kyoto Encyclopedia of Genes and Genomes) is the major public pathway-related database (http://www.genome.jp/kegg/). We employed KOBAS software to perform pathway and functional classification of oilseed rape pods, and analyzed all DEGs by mapping to the KEGG database. To know the significant terms of the DEGs, the KO enrichment analyses of up-regulated and down-regulated genes of HFA15 vs HFA25, LFA15 vs LFA25, LFA15 vs HFA15 and LFA25 vs HFA25 were completed against the background of KO term distribution of all expression genes. The most enriched twenty KO terms are shown in Fig 3. The most enrichment DEGs KO terms in both high oil content line and low oil content line between 15 DAP and 25 DAP are metabolic pathways. Furthermore, the most significantly different two DEGs KO terms are photosynthesis-antenna proteins and photosynthesis. These results revealed the different metabolic levels in the different stages of pod development, especially the photosynthesis related to starch and sugar metabolism, and fatty acid metabolism (Fig 3A and 3B, S2 and S3 Tables). So we then focus on the analysis of the significant KO terms of the DEGs between the two varieties at lipid synthetic critical period (25 DAP).

Additionally, pyruvate metabolism, glycolipid metabolism, glyceophospholipid metabolism, fatty acid biosynthesis, fatty acid elongation, ether lipid metabolism, sphingolipid metabolism, and especially the starch and sugar metabolism are the most enrichment DEGs KO terms between the two oilseed rape lines at 25 DAP (Fig 3D and S5 Table). On the other hand, the most enrichment DEGs KO terms of LFA15 vs HFA15 is ribosome (Fig 3C and S4 Table). Beyond that, the number of DEGs of LFA25 vs HFA25 related to the starch and sugar metabolism, pyruvate metabolism and fatty acid biosynthesis is significantly more than that of LFA15 vs HFA15.

### Analysis of DEGs related to photosynthesis, starch and sugar metabolism, and pyruvate metabolism

**Fig 4:**
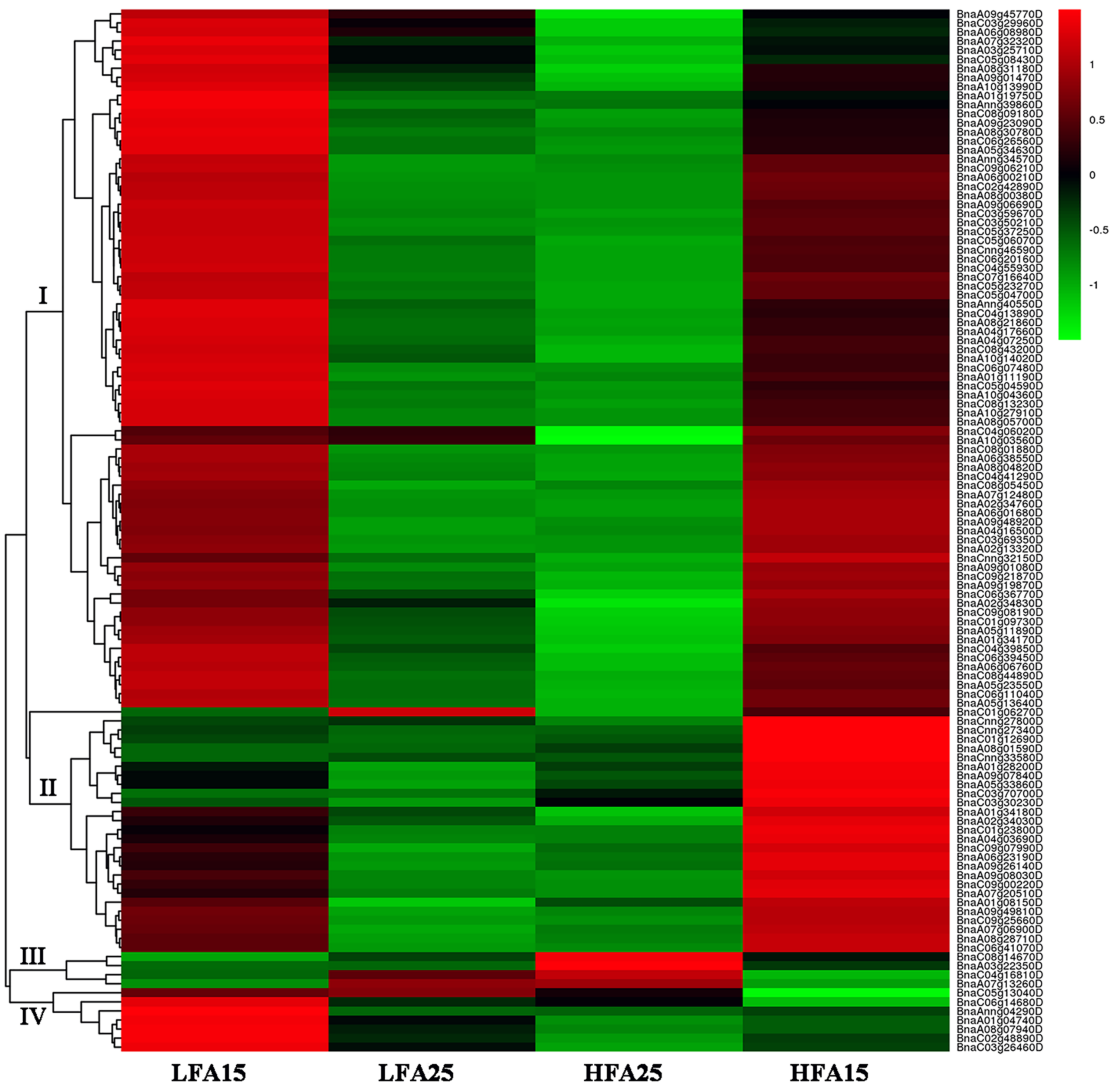
Hierarchical clustering analysis of the differentially expressed genes (DEGs) related to photosynthesis.

**Fig 5:**
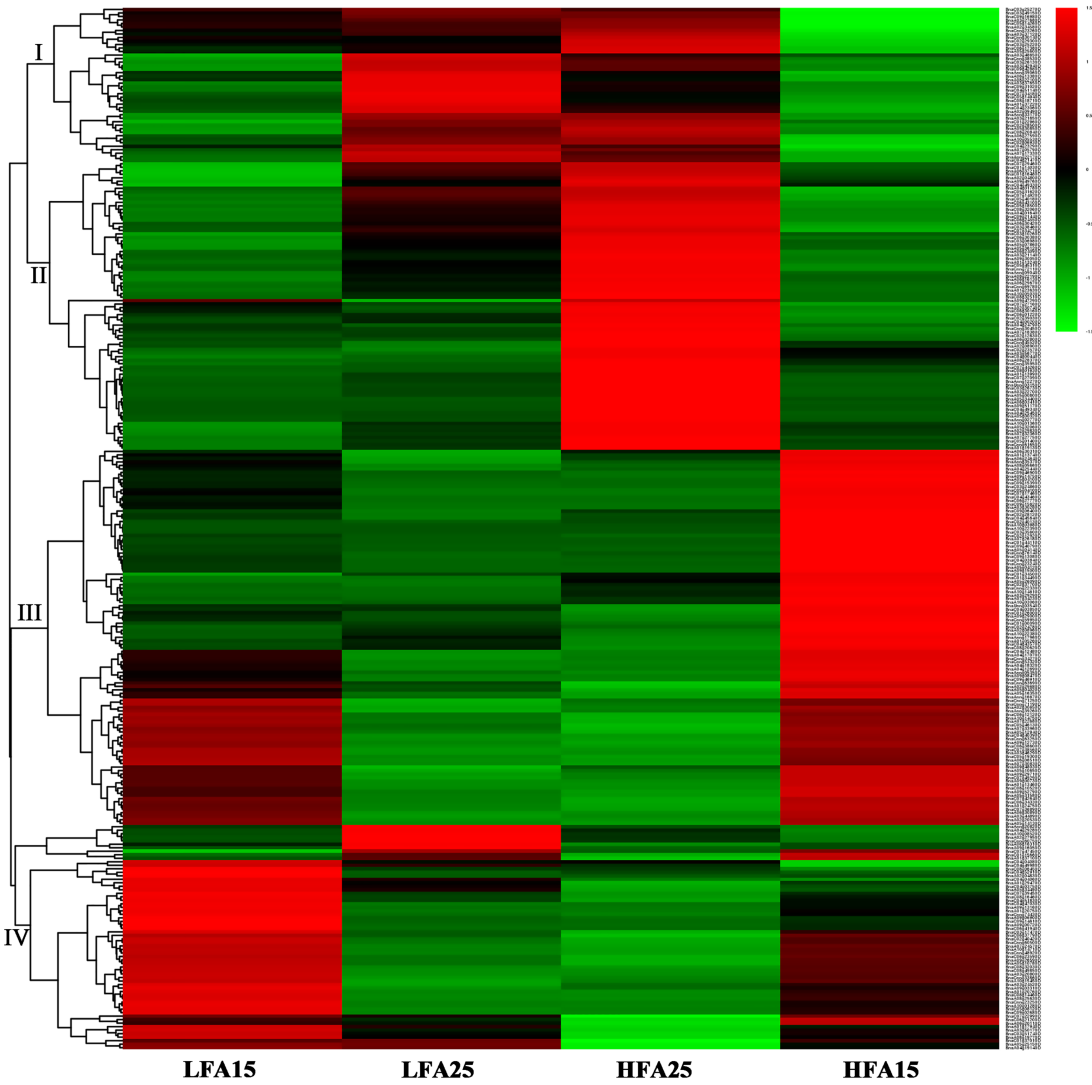
Hierarchical clustering analysis of the differentially expressed genes (DEGs) related tostarch and sugar metabolism.

**Fig 6:**
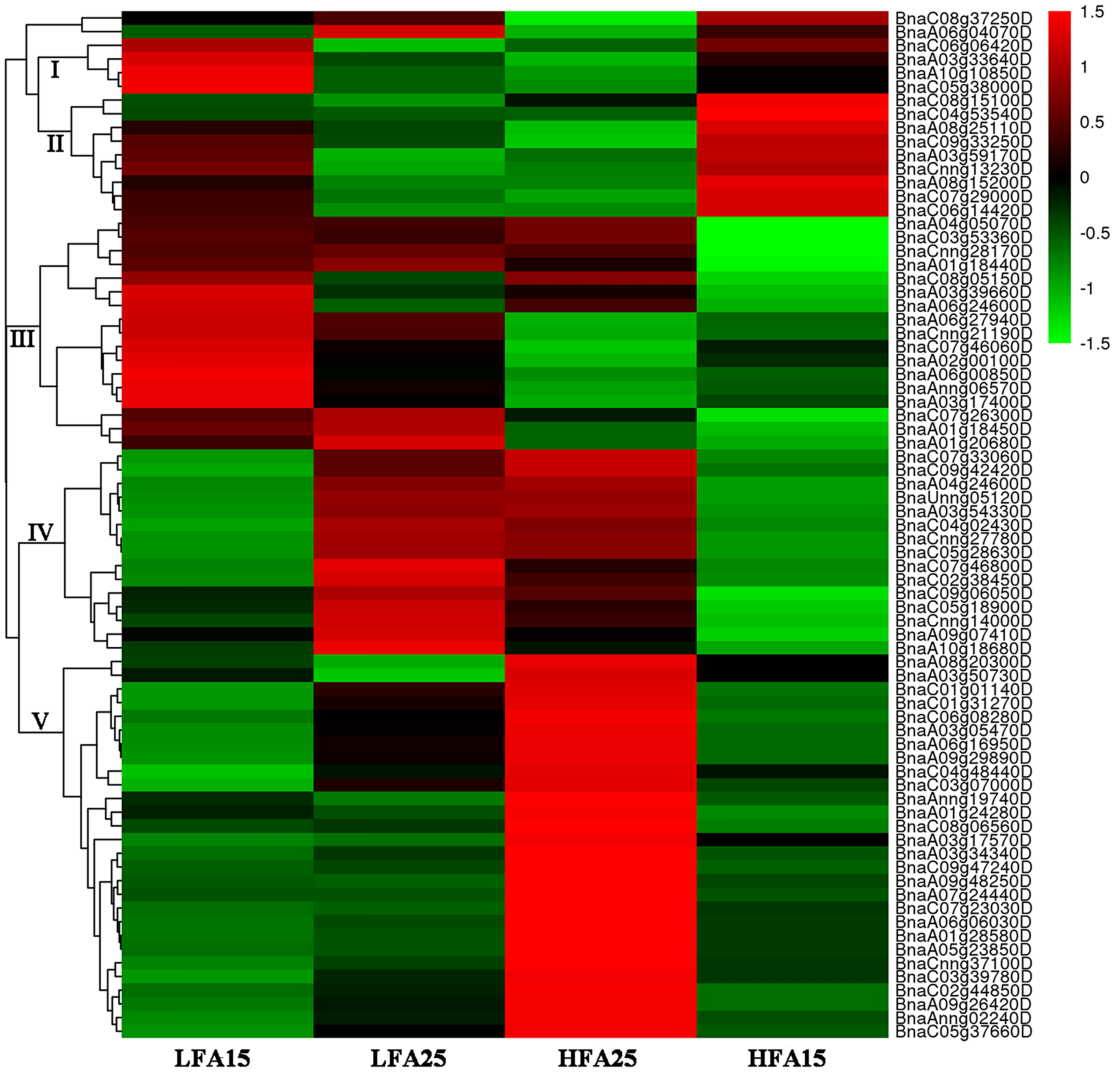
Hierarchical clustering analysis of the differentially expressed genes (DEGs) related to pyruvate metabolism.

In addition to the protective function of encapsulating from pathogens and pest, the photosynthate from silique walls contributes nutrients to fuel seed growth and oil accumulation of oilseed rape [36]. Moreover, the GO enrichment analysis of DEGS and the KEGG pathway enrichment analysis revealed that the expression of the genes related to photosynthesis, starch and sugar metabolism, and pyruvate metabolism is significantly different in two kinds of siniques at 15 DAP and 25 DAP. To explore the expression patterns of the genes related to photosynthesis, starch and sugar metabolism, and pyruvate metabolism, we performed hierarchical clustering of all DEGs in these pathways, and used Pearson correlation to determine the distance metric for gene expression patterns with functional enrichment. In addition, log ratio values (FPKM) were used for gene expression analysis. The hierarchical clustering analysis of the genes related to photosynthesis indicates that the expression of the most genes at 15 DAP is significantly higher than that at 25 DAP (Fig 4). However, some genes (class III) show different expression patterns. Namely, the expression of them at 25 DAP is significantly higher than that at 15 DAP, and the expression of them in siliques of high oil content lines is higher than that in low oil content lines. These genes may play important roles in affecting the oil content at 25 DAP and may be potential targets for genetically engineering improved oil content. Although showing similar expression patterns at 25 DAP, the expression of some genes (class IV), which may affect the oil content, in high oil content materials at 15 DAP is higher than that in low oil content materials (Fig 4). Moreover, the hierarchical clustering analysis of the genes related to starch/sugar metabolism and pyruvate metabolism show that the expression of most of the genes in high oil content lines is higher than that in low oil content lines, especially the genes (such as *BnaA09g26420D*, *BnaAnng02240D*, *BnaA03g34340D*, *BnaC03g39780D*, *BnaC07g23030D* and so on) involved in glycolytic pathway and pyruvate dehydrogenase complex (Figs 5 and 6). Additionally, expression of the genes related to de novo fatty acid biosynthesis in high oil content materials is also higher than those in low oil content materials, and some of these genes were up-regulated in siliques at later developmental stage (S3 Fig). The above results indicate that the genes related to photosynthesis, starch and sugar metabolism, and pyruvate metabolism have a significant impact on pod development and oil content of *B*. *napus*.

### The role of oil content-related transcription factors (TFs) in pod wall development and oil accumulation

By far, only few studies have been done to investigate transcription factors (TFs) and their target genes involved in oil accumulation in *B*. *napus*. Here, the GO enrichment analysis of DEGS shows that DNA binding (GO:0051090) is one of the predominant subcategories of HFA15 vs HFA25, LFA15 vs LFA25, LFA15 vs HFA15, and LFA25 vs HFA25, which indicates TFs may not only have an effect on seed development and oil accumulation but also play an important role in pod wall metabolism.

**Fig 7:**
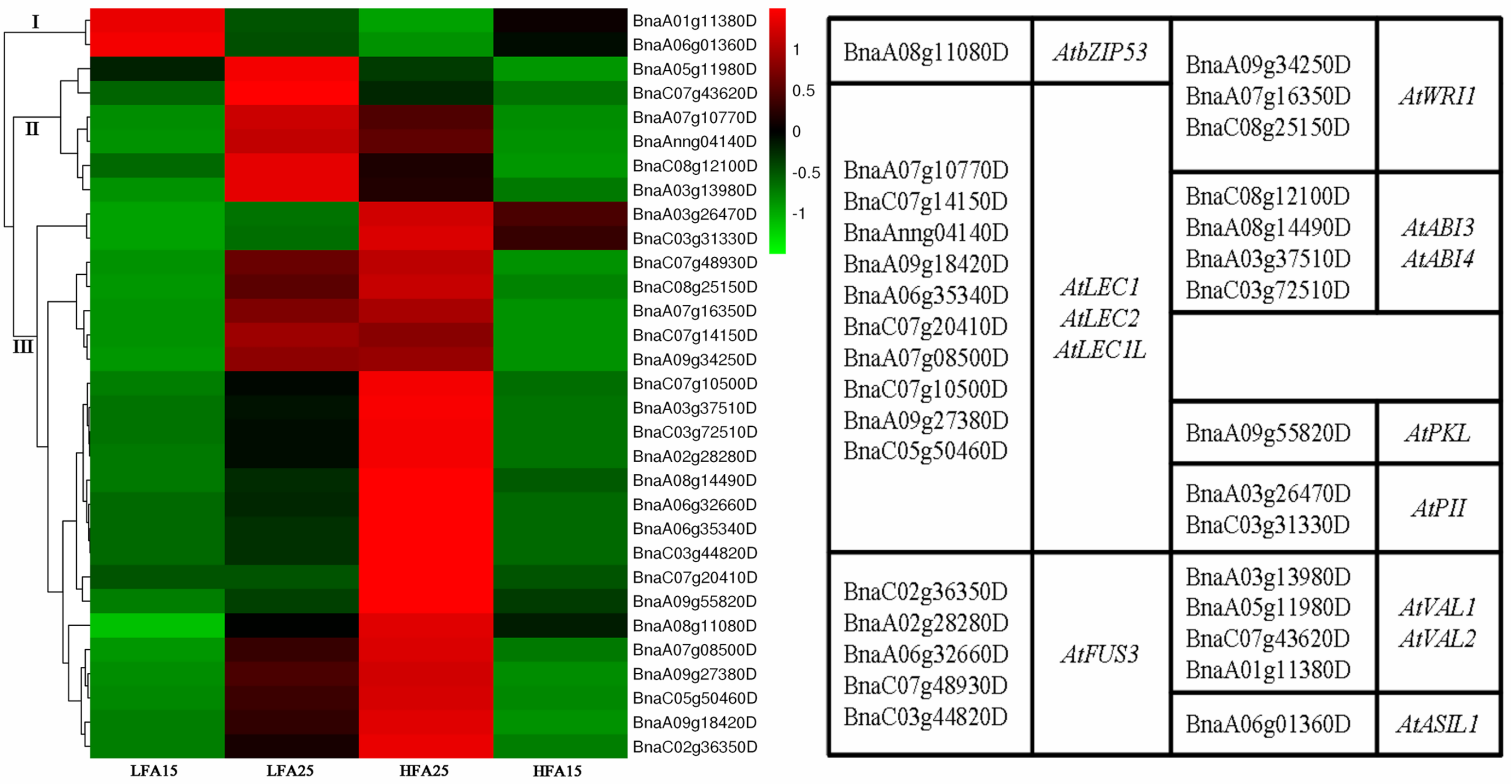
Hierarchical clustering analysis of the differentially expressed genes (DEGs) encoding transcription factors (TFs).

**Fig 8:**
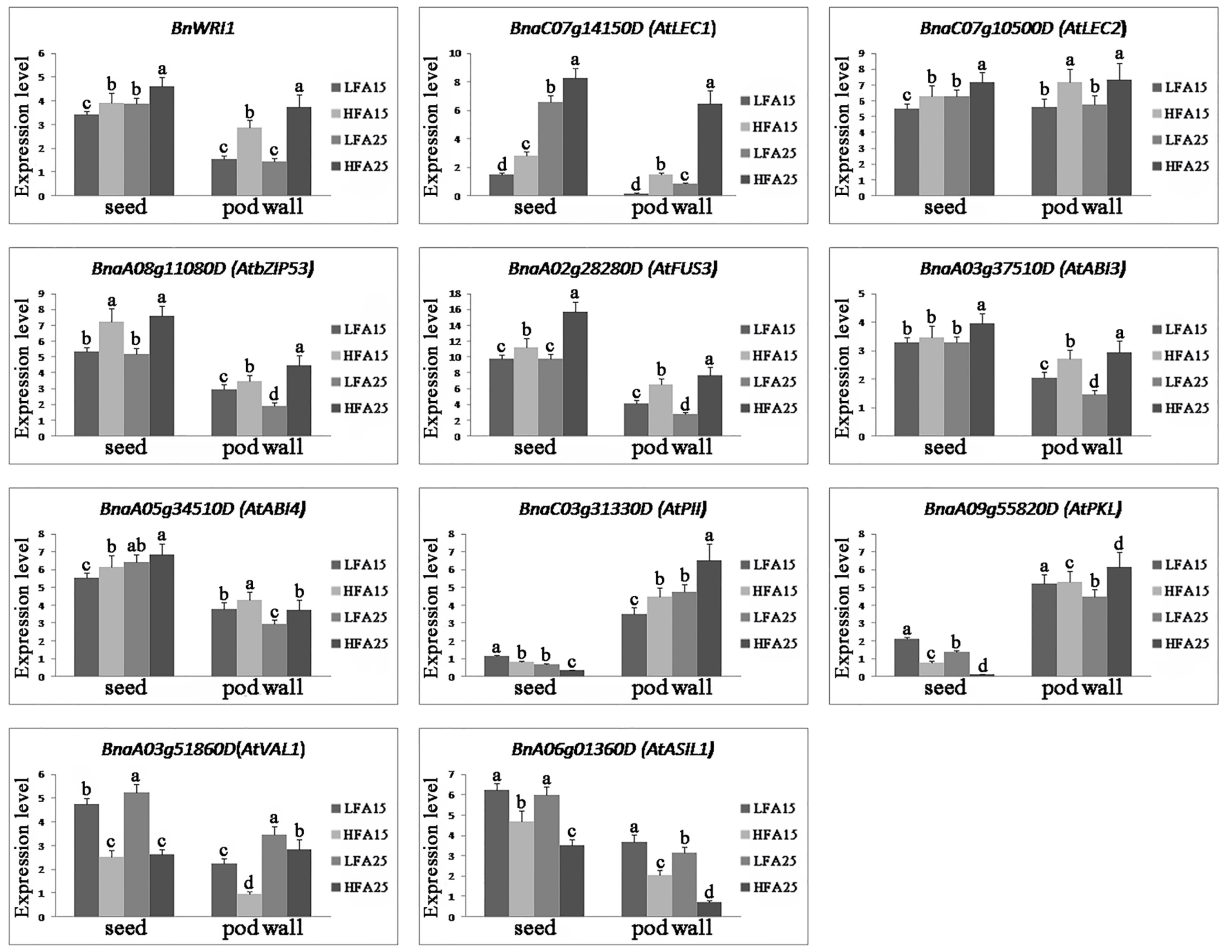
Quantitative RT-PCR analysis of expression of the transcription factor (TF) genes. The expression of the TF genes was analyzed in pod walls and seeds. The gene expression level refers natural logarithm of the expression value. The results were the average of three biological replicate samples in triplicate, and error bars indicate the standard errors. Significance of difference was analyzed by Duncan’s test (P<0.05).

To analyze the role of TFs related to oil content in pods, the *B*. *napus* homologs of some *Arabidopsis* TF genes (including positive and negative regulators in FA synthesis and TAG assembly) were searched from the data of DNA binding subcategory. Through basic local alignment search tool (blast), we identified 31 differentially expressed TFs. The hierarchical clustering analysis of these TFs shows that the expression of the positive regulators in HFA15 and HFA25 was higher than that in LFA15 and LFA25, which is in stark contrast to negative regulators. In *B*. *napus*, the homologs of *AtLEC1*, *AtABI3*, *AtFUS3*, *AtWRI1*, and *AtbZIP53* are up-regulated with the increased oil content, and the homologs of *AtVAL1* and *AtASIL1* are down-regulated with the decreased oil content (Fig 7). However, whether these TFs affect pod wall metabolism and seed oil accumulation are still unclear. Hence, qRT-PCR was performed to examine the transcript levels of these TFs in seeds and pod walls at 15 and 25 DAP respectively. As shown in Fig 8, the transcriptional levels of *BnWRINKLED1*, *BnaC07g14150D*, *BnaC07g10500D*, *BnaA08g11080D*, *BnaA02g28280D*, *BnaA03g37510D*, *BnaA05g34510D*, *BnaA03g5180D*, and *BnA06g01360D* in seeds and pod walls were consistent with the RNA-seq results. The results suggest that the TFs indeed not only have an effect on seed development and oil accumulation but also play an important role in pod wall metabolism. It is interesting that the expression of *BnaC03g31330D* and *BnaA09g55820D* in seeds and pot walls is different. The transcription levels of *BnaC03g31330D* and *BnaA09g55820D* in pod walls of HFA15 and HFA25 were higher than those in LFA15 and LFA25, consistent with the RNA-Seq results. However, the transcription levels of *BnaC03g31330D* and *BnaA09g55820D* in seeds of HFA15 and HFA25 were lower than those in LFA15 and LFA25, suggesting that *BnaC03g31330D* and *BnaA09g55820D* may play different roles in pod walls and in seeds (Fig 8).

Meanwhile, we examined the transcription levels of photosynthesis related genes, *BnRBCS1A* and *BnRBCS3B*, in pod walls and BnWRI1 downstream gene *BnBCCP2* (encoding biotin carboxyl carrier protein) in seeds (S4 Fig). All of the three genes showed higher expression levels in HFA15 and HFA25 than those of LFA15 and LFA25.

From our data, on the one hand, *BnWRINKLED1*, *BnaA03g51860D*, *BnaC07g14150D*, *BnaC07g10500D*, *BnaA08g11080D*, *BnaA02g28280D*, *BnaA03g37510D*, *BnaA05g34510D*, *BnaC03g31330D*, *BnaA09g55820D*, and *BnA06g01360D* may be the upstream regulators for modulating the genes involved in *de novo* fatty acid synthesis and triacylglycerol assembly at different developmental stages. On the other hand, these TFs may also regulate the genes involved in photosynthesis, starch and sugar metabolism, and pyruvate metabolism, which are crucial for oil accumulation.

## Discussion

### Comparative analysis of gene expression between different stages and varieties

The close relationship between silique and seed development in *B*. *napus* determines the crucial function of silique in seed yield and quality establishment [46, 47]. Moreover, silique is the sole tissue that directly connects with seed through funiculus. It not only protect seed encapsulating from pest and pathogens but also acts as a source of nutrients for seed growth [48, 49]. Up to now, nearly all studies on oil content have focused on *de novo* fatty acid synthesis and triacylglycerol assembly pathway in seeds [37, 40]. However, these studies have ignored the role of other tissues that may affect oil content. This lack of understanding may hinder the development of future plant breeding [37, 40–43]. Thus, we performed RNA sequencing technique (RNA-Seq) to explore the molecular mechanism of oil-related biological processes in siliques of two *B*. *napus* lines with low and high oil content respectively at 15 and 25 DAP. A large number of differentially expressed genes (DEGs) involved in multiple pathways were identified among these samples through the comparative analysis of their transcriptomes. Quantitative RT-PCR analysis showed that the relative expression patterns of the genes were consistent with RNA-Seq data, demonstrating that the RNA-Seq data are reliable (S2 Fig). The GO analysis showed that the DNA binding was one of the most significantly differential GO subcategories among the four samples, which indicates that TFs play an important role in pod development. ATP binding that involved in energy metabolism and oxidation-reduction process is one of the significant DEG GO subcategories among the four samples, which shows that the energy metabolism levels of siliques are different between the two rape materials at different developmental stages and have a great effect on silique development and seed oil accumulation.

Some DEGs related to metabolic pathways, especially the photosynthesis-antenna proteins and photosynthesis, pyruvate metabolism, glycolipid metabolism, glyceophospholipid metabolism, fatty acid biosynthesis, fatty acid elongation, ether lipid metabolism, sphingolipid metabolism, and starch and sugar metabolism exhibited different expression patterns among these four samples. These metabolic pathways are important for the pod development, and then may further affect the oil accumulation in seeds.

### The role of the genes related to photosynthesis, starch and sugar metabolism, and pyruvate metabolism in oil accumulation

Silique wall photosynthesis plays an important role in regulation of seed oil content in terms of maternal effects [37]. A study found that the increased brassinosteroid level in maternal tissues of rice led to enhanced photosynthetic efficiency and increased assimilation of glucose to starch in seeds [50]. Conversion of carbohydrates into lipids, proteins and secondary metabolites is an elaborate regulation system during seed development, which greatly affects seed oil content [51, 52]. Similarly, our data also revealed the important role of maternal effect in seed oil content, consistent with the previous studies. Additionally, we found that a lot of genes were up-regulated in 25 DAP siliques of the high oil content lines, suggesting they may be involved in regulating the oil content in seeds. Meanwhile, the genes that show similar expression patterns at 25 DAP but were up-regulated in high oil content materials at 15 DAP may affect the pod metabolism and the oil content at 15 DAP.

Moreover, the hierarchical clustering analysis showed that expressions of the genes related to starch and sugar metabolism, pyruvate metabolism, and de novo fatty acid biosynthesis in high oil content materials were higher than those in low oil content materials, and most of these genes were up-regulated in siliques at later developmental stage (Figs 4–6 and S3 Fig), especially the genes involved in glycolytic pathway and pyruvate dehydrogenase complex. For example, *BnaA03g34340D*, *BnaC03g39780D* and *BnaC07g23030D*, which are key genes related to the pathway of pyruvate converted into acetyl-CoA, were up-regulated at the later stage, especially in the high oil content materials. We also found that many lipid metabolism-related genes involved in glyceolipid metabolism, glyceophospholipid metabolic, fatty acid biosynthesis, fatty acid elongation, ether lipid metabolism, sphingolipid metabolism were differentially expressed among the four samples, especially between HFA25 and LFA25. Given the data together, our results suggest that expression profiling of the genes related to photosynthesis, starch and sugar metabolism, pyruvate metabolism, and lipid metabolism in different oilseed rape lines at different developmental stages may result in differential oil accumulation in seeds.

### Transcription factors (TFs) related to oil content play important roles in silique and seed development

It’s worth noting that several recent studies in maize and *Arabidopsis thaliana* have delineated a complex network of transcription factors that control the gene expression programs essential to accomplish seed maturation [53]. A wide set of TFs were thus shown to control the transition between vegetative phases of development and seed maturation [54]. However, the regulation and relative contribution of silique photosynthesis, starch and sugar metabolism, pyruvate metabolism, and lipid metabolism remain largely unclear so far.

**Fig 9:**
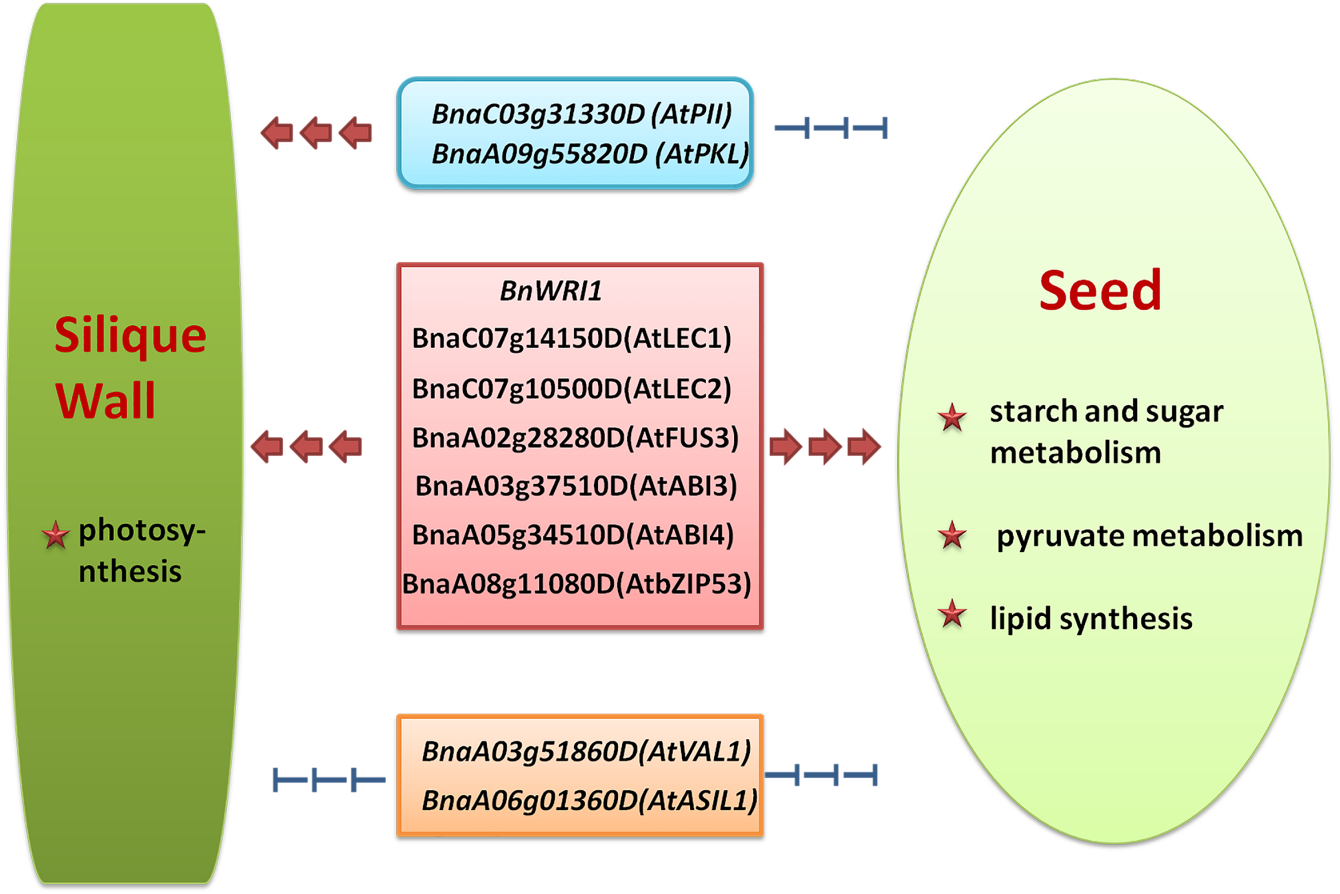
Overview of the roles of the oil content-related transcription factors (TFs) in silique wall and seed metabolism.

It has been reported that *BnGRF2* enhances seed oil production through regulating cell number and plant photosynthesis [55]. Furthermore, sucrose may also play a role in triggering the induction of WRI1 [24]. In this study, our results indicated there may be a complex feedback regulation network for TFs regulating photosynthesis, starch and sugar metabolism, pyruvate metabolism, and lipids synthesis. More intriguingly, the transcription levels of *BnaC03g31330D* and *BnaA09g55820D* in pod wall of HFA15 and HFA25 were higher than those of LFA15 and LFA25. However, the transcription levels of *BnaC03g31330D* and *BnaA09g55820D* in seeds of HFA15 and HFA25 were lower than those of LFA15 and LFA25. The different expression levels of these TF genes imply that the different TFs may play different roles in pod wall and seed development. Combined the expression patterns of the TFs (such as *BnWRINKLED1*, *BnaC07g14150D*, *BnaC07g10500D*, *BnaA08g11080D*, *BnaA02g28280D*, *BnaA03g37510D* and *BnaA05g34510D*, which are homologs of *AtWRI1*, *AtLEC1*, *AtLEC2*, *AtbZIP53*, *AtFUS3*, *AtABI3* and *AtABI4*, respectively) with the genes related to photosynthesis, starch and sugar metabolism, pyruvate metabolism, and lipid metabolism, we hypothesize that these TFs may positively regulate the silique metabolism and seed oil accumulation. On the other hand, *BnaA06g01360D* and *BnaA03g51860D* may act as negative regulators in controlling expression of the genes related to photosynthesis, starch and sugar metabolism, pyruvate metabolism, and lipid metabolism (Fig 9).

In conclusion, a comprehensive characterization of DEGs (including TFs) related to photosynthesis, starch and sugar metabolism, pyruvate metabolism, and lipid synthesis in siliques of two *B*. *napus* lines with low and high oil contents respectively at 15 and 25 DAP by the RNA-Seq technique has generated new data relating to the oil accumulation in seeds. Thus, the knowledge acquired in this study provides new rational targets for future work on the molecular mechanism of seed oil formation and molecular breeding of *B*. *napus*.

## Supporting information

S1 Dataset List of the 944 novel transcripts detected from the four cDNA libraries. (XLS)

S2 Dataset Overview of all GO terms in HFA15-vs-HFA25. (XLS)

S1 Fig Expressions of *BnBCCP*, *BnWRI1* and *BnFAD* genes in seeds and seed morphology of *Brassica napus* HFA and LFA lines at different developmental stages. (PDF)

S2 Fig Quantitative RT-PCR analysis to validate expression of the selected differentially expressed genes (DEGs) in siliques of *B*. *napus* lines (LFA15, LFA25, HFA15 and HFA25). (PDF)

S3 Fig Hierarchical clustering analysis of differentially expressed genes (DEGs) related to de novo fatty acid biosynthesis. (PDF)

S4 Fig Quantitative RT-PCR analysis of gene expression in seeds and pod walls of *B*. *napus*. (PDF)

S1 Table Primers used in real-time quantitative RT-PCR analysis. (PDF)

S2 Table Overview of the most enrichment of 30 different expression KEGG pathways in HFA15-vs-HFA25. (PDF)

S3 Table Overview of the most enrichment of 30 different expression KEGG pathways in LFA15-vs-LFA25. (PDF)

S4 Table Overview of the most enrichment of 30 different expression KEGG pathways in HFA15-vs-LFA15. (PDF)

S5 Table Overview of the most enrichment of 30 different expression KEGG pathways in HFA25-vs-LFA25. (PDF)
