# Supplementary material for: Transcriptome profiling analysis reveals the role of silique in controlling seed oil content in Brassica napus
Source: PLoS One. 2017 Jun 8;12(6):e0179027. doi: 10.1371/journal.pone.0179027 (PMC5464616; doi:10.1371/journal.pone.0179027)
Supplement: S1 Fig — (PDF) [file pone.0179027.s003.pdf]

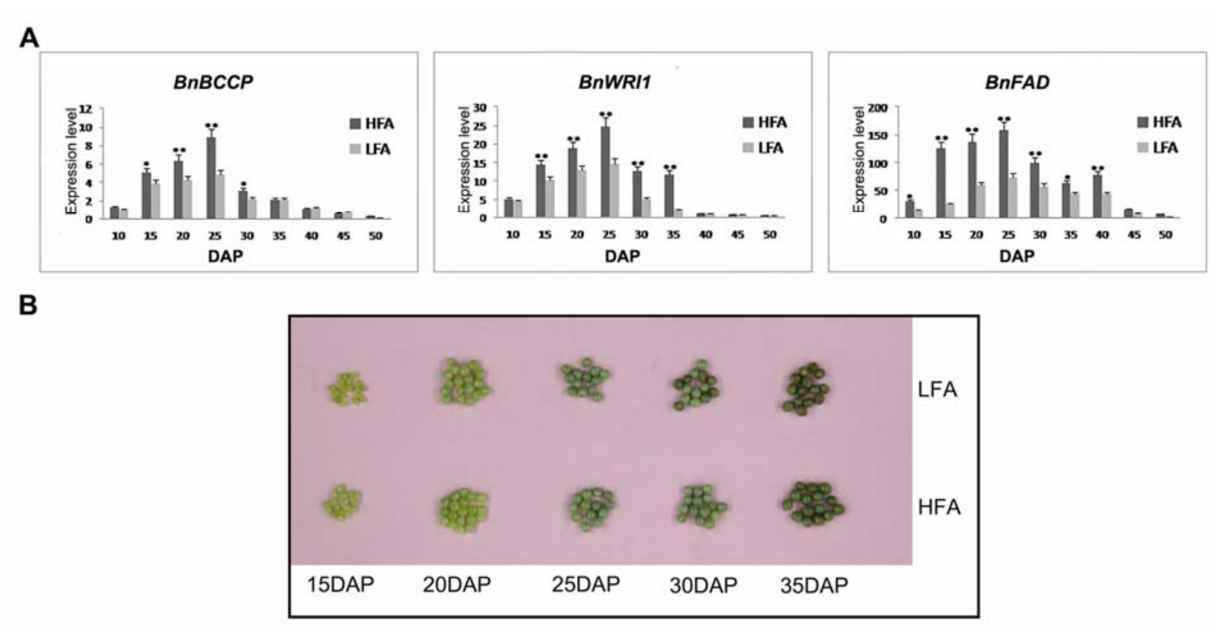

S1 Fig. Expressions of *BnBCCP*, *BnWRI1* and *BnFAD* genes in seeds and seed morphology of *Brassica napus* HFA and LFA lines at different developmental stages. (**A**) Quantitative RT-PCR analysis of expression of *BnBCCP*, *BnWRI1* and *BnFAD* in seeds *B. napus*. The expression of BnBCCP, BnWRI1 and BnFAD were analyzed in seeds at different developmental stages. The gene expression level refers natural logarithm of the expression value. The results were the average of three biological replicate samples in triplicate, and error bars indicate the standard errors. Significance of difference between HFA and LFA was analyzed by Duncan's test (*. P<0.05; **. P<0.01). (**B**) Comparison of seed morphology of HFA and LFA at different developmental stages. HFA, high oil content line of *B. napus*; LFA, low oil content line of *B. napus*.
