# Supplementary material for: Transcriptome profiling analysis reveals the role of silique in controlling seed oil content in Brassica napus
Source: PLoS One. 2017 Jun 8;12(6):e0179027. doi: 10.1371/journal.pone.0179027 (PMC5464616; doi:10.1371/journal.pone.0179027)
Supplement: S2 Fig — (PDF) [file pone.0179027.s004.pdf]

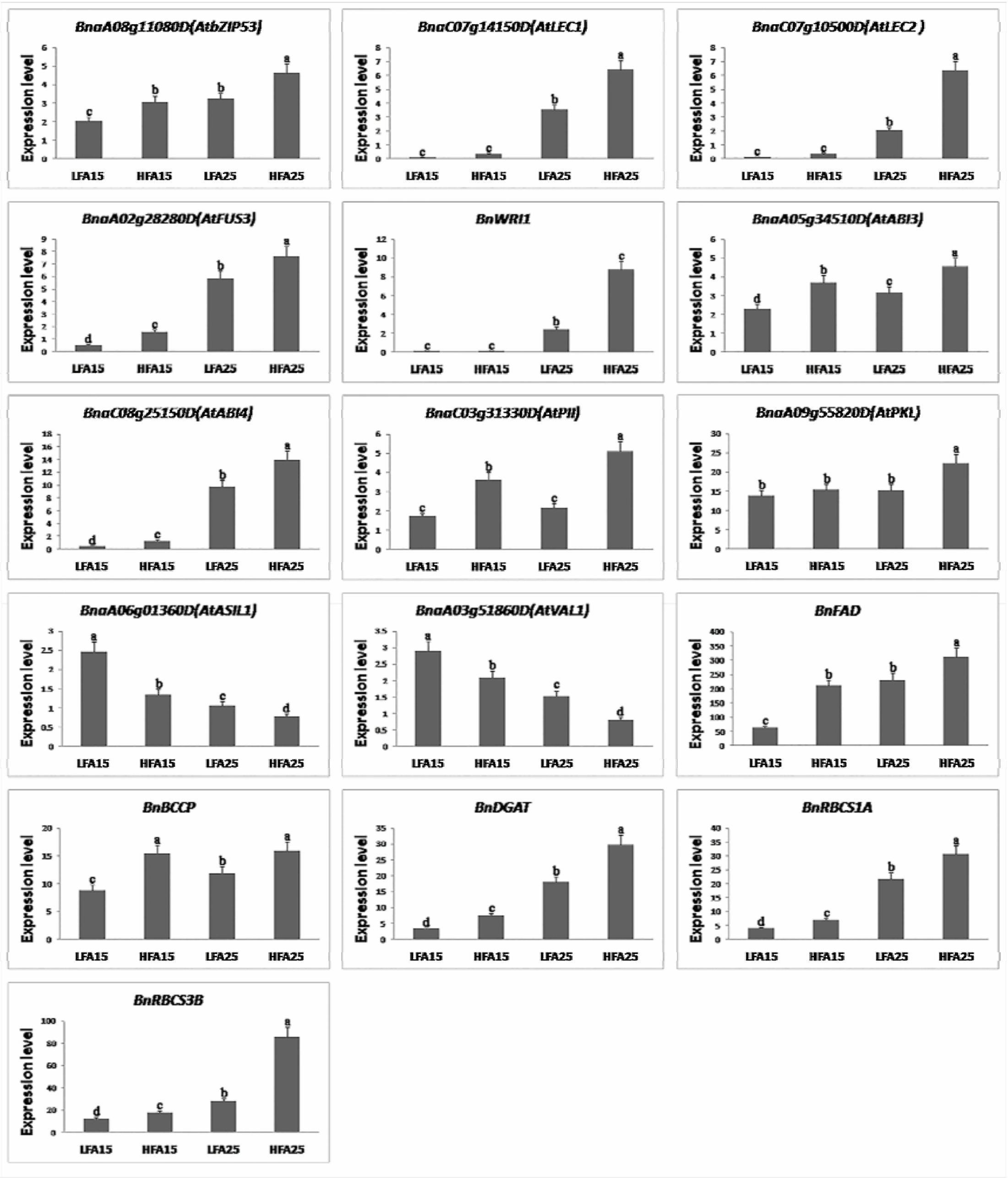

S2 Fig. Quantitative RT-PCR analysis to validate expression of the selected differentially expressed genes (DEGs) in siliques of *B. napus* lines (LFA15, LFA25, HFA15 and HFA25). The gene expression level refers natural logarithm of the expression value. The results were the average of three biological replicate samples in triplicate, and error bars indicate the standard errors. Significance of difference was analyzed by Duncan’s test ($P<0.05$).
