# Supplementary material for: Transcriptome profiling analysis reveals the role of silique in controlling seed oil content in Brassica napus
Source: PLoS One. 2017 Jun 8;12(6):e0179027. doi: 10.1371/journal.pone.0179027 (PMC5464616; doi:10.1371/journal.pone.0179027)
Supplement: S3 Fig — (PDF) [file pone.0179027.s005.pdf]

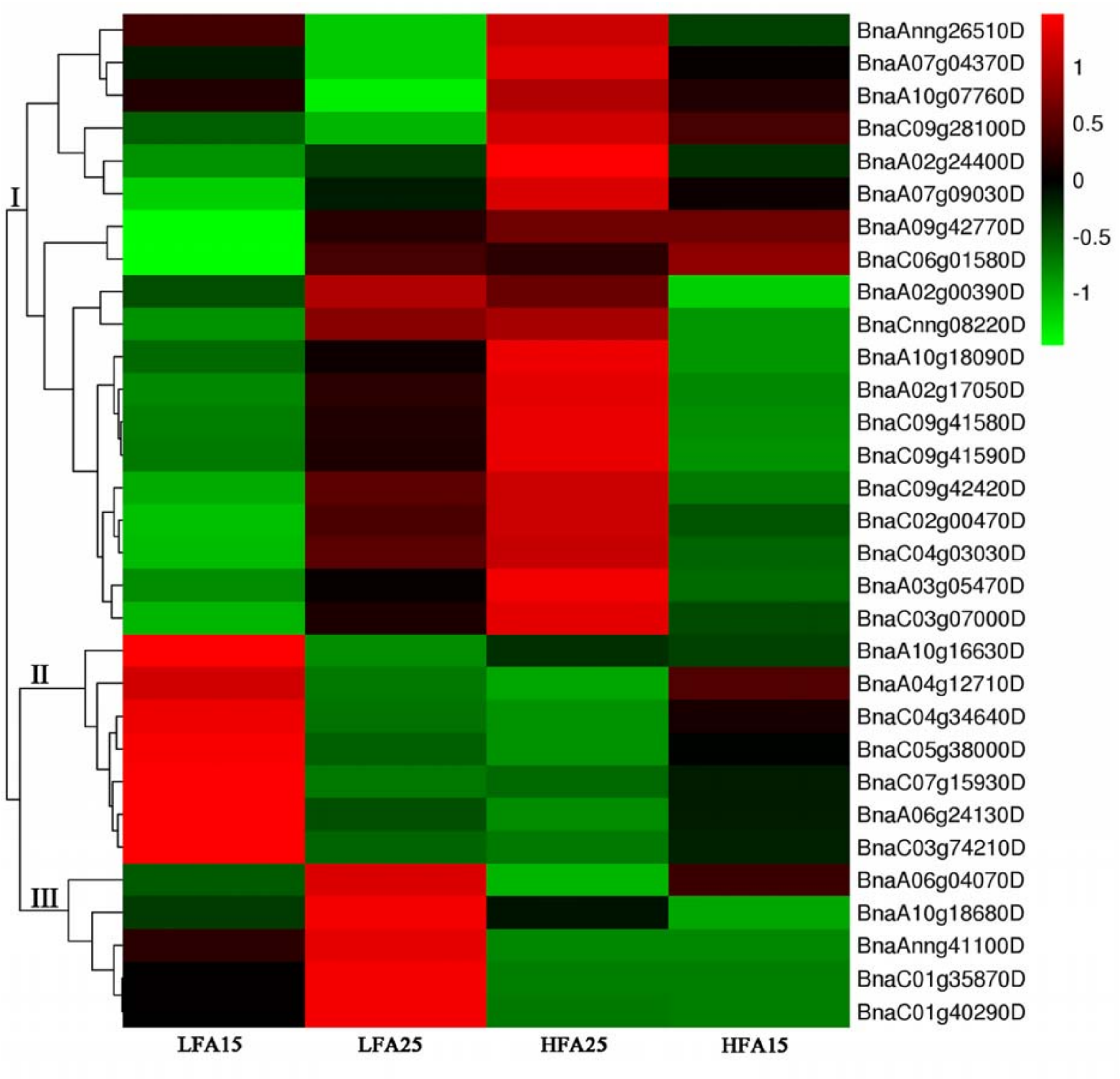

S3 Fig. Hierarchical clustering analysis of differentially expressed genes related to de novo fatty acid biosynthesis.
