# Supplementary material for: Transcriptome profiling analysis reveals the role of silique in controlling seed oil content in Brassica napus
Source: PLoS One. 2017 Jun 8;12(6):e0179027. doi: 10.1371/journal.pone.0179027 (PMC5464616; doi:10.1371/journal.pone.0179027)
Supplement: S4 Fig — (PDF) [file pone.0179027.s006.pdf]

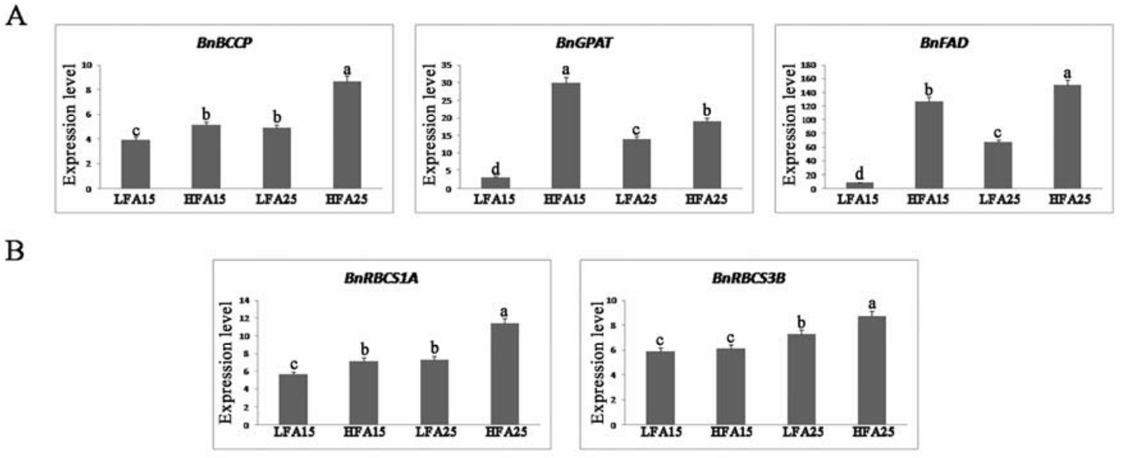

S4 Fig. Quantitative RT-PCR analysis of gene expression in seeds and pod walls of *B. napus*. (A) qRT-PCR analysis of *BnBCCP*, *BnGPAT* and *BnFAD* in seeds. (B) qRT-PCR analysis of *BnRBCS1A* and *BnRBCS3B* in pod walls. The gene expression level refers natural logarithm of the expression value. The results were the average of three biological replicate samples in triplicate, and error bars indicate the standard errors. Significance of difference was analyzed by Duncan's test ($P<0.05$).
