# Supplementary material for: Transcriptome profiling analysis reveals the role of silique in controlling seed oil content in Brassica napus
Source: PLoS One. 2017 Jun 8;12(6):e0179027. doi: 10.1371/journal.pone.0179027 (PMC5464616; doi:10.1371/journal.pone.0179027)
Supplement: S1 Table — (PDF) [file pone.0179027.s007.pdf]

S1 Table. Primers used in real-time quantitative RT-PCR analysis

primer names	sequence (5’-3’)
BnACT	GACCGTATGAGCAAAGAGATCACA
	TCTCGGGAGGTGCAACGA
BnBCCP	AACTTTATGGCTAAAGTTTCAGGTCTTC
	GCTTAAGTTCAAGTTCCACTATATCTCTTG
BnWRI1	GCTGAAACCAAACAAGAAGTGGAAGCTAAA
	CTCAGTCGAAGGCAAACCAT
*Bn*FAD	TCTCATCTTCCACTCCTTCC
	CAAACACTTCGTCTCTCTCG
BnDGAT	TGGCGGGTACTTTTGGTATC
	GGCGTATTTCATGGTTGCTC
BnA03g3751ODRT	ATGCATGGGATCTCGTGGAC
	ACTGTAACGATCTCCCCTGA
BnA05g3451ODRT	CCAATAGCTCACAACTCGGC
	GCTCGTTTCCTAGCAGCTTC
BnA06g0136ODRT	TTGGGTTCACTGAGGCGTAC
	TCAGCTACTTACATCGCCATT
BnA08g1108ODRT	ATGGGGCCGTTTCTTTTGTC
	TGCCAACAGAGCCTTTATTACA
BnA02g2828ODRT	TTCGAGGGTTTTGGCTCAGT
	CGAAATTGGTTTGCTTTTAC
BnC07g1415ODRT	AAGTGGTGGTGGTGGATCTT
	CTGAAACTGAAGTTTGATATTC
BnC07g1050ODRT	TGGATCTCACTGTCGATCTTGA
	TCTGGAGCCAAACACAAATAAGT
BnC03g3133ODRT	ACAAGAGCTTAAGAATGGCGT
	GGACAAGGACATGAAACAGGG
BnA09g5582ODRT	AGAAGCTCAGTGTCCGTGAT
	GGAACGGCTAAGTAAAATGACAG
BnA03g5186OD RT	GCCTGAGCCAATGAACTAACT
	CCTCACCAATATTCTTTTCTCTCTC
BnRBCS3B	GACATTACTTCCATCGCAAGCAA
	TGATCCTGATGAATGCGTTAGGG
BnRBCS1A	AAGCTTCCCTTGTTCGGTTGCAC
	GCTTGGTGGCTTGTAGGCAATGA
