# Supplementary material for: Transcriptome profiling analysis reveals the role of silique in controlling seed oil content in Brassica napus
Source: PLoS One. 2017 Jun 8;12(6):e0179027. doi: 10.1371/journal.pone.0179027 (PMC5464616; doi:10.1371/journal.pone.0179027)
Supplement: S2 Table — (PDF) [file pone.0179027.s008.pdf]

S2 Table. Overview of the most enrichment of 30 differential expression KEGG pathways in HFA15-vs-HFA25

Pathway	Up-regulated genes number	Down-regulated genes number
Photosynthesis	6	109
Photosynthesis - antenna proteins	3	46
Phenylpropanoid biosynthesis	123	115
Phenylalanine metabolism	77	53
Starch and sucrose metabolism	136	161
Ascorbate and aldarate metabolism	37	42
Biosynthesis of secondary metabolites	633	549
Metabolic pathways	953	1114
Phenylalanine, tyrosine and tryptophan biosynthesis	30	28
Linoleic acid metabolism	10	19
Indole alkaloid biosynthesis	21	14
alpha-Linolenic acid metabolism	38	35
Arginine and proline metabolism	53	40
Glutathione metabolism	46	40
Pentose and glucuronate interconversions	62	90
Isoquinoline alkaloid biosynthesis	14	14
Tropane, piperidine and pyridine alkaloid biosynthesis	19	20
Nitrogen metabolism	19	36
Fatty acid biosynthesis	35	6
Glyoxylate and dicarboxylate metabolism	25	44
Flavonoid biosynthesis	77	39
Glycerolipid metabolism	38	21
Monoterpenoid biosynthesis	3	8
Propanoate metabolism	26	14
Circadian rhythm - plant	64	46
Arachidonic acid metabolism	13	9
Cysteine and methionine metabolism	47	37
Sphingolipid metabolism	19	21
Inositol phosphate metabolism	30	38
Other types of O-glycan biosynthesis	10	6
