# Supplementary material for: Transcriptome profiling analysis reveals the role of silique in controlling seed oil content in Brassica napus
Source: PLoS One. 2017 Jun 8;12(6):e0179027. doi: 10.1371/journal.pone.0179027 (PMC5464616; doi:10.1371/journal.pone.0179027)
Supplement: S3 Table — (PDF) [file pone.0179027.s009.pdf]

S3 Table. Overview of the most enrichment of 30 different expression KEGG pathways in LFA15-vs-LFA25

Pathway	Up-regulated genes number	Down-regulated genes number
Photosynthesis - antenna proteins	3	45
Photosynthesis	4	102
Phenylpropanoid biosynthesis	143	101
Starch and sucrose metabolism	117	191
Ribosome biogenesis in eukaryotes	137	12
Glyoxylate and dicarboxylate metabolism	28	61
Phenylalanine metabolism	79	45
Phenylalanine, tyrosine and tryptophan biosynthesis	41	18
Indole alkaloid biosynthesis	20	15
Glutathione metabolism	59	40
Cysteine and methionine metabolism	69	35
Ascorbate and aldarate metabolism	32	41
Circadian rhythm - plant	68	58
Linoleic acid metabolism	6	19
Vitamin B6 metabolism	11	4
alpha-Linolenic acid metabolism	33	40
Nitrogen metabolism	19	39
Cyanoamino acid metabolism	35	47
Biosynthesis of secondary metabolites	640	568
Arginine and proline metabolism	58	34
Pentose and glucuronate interconversions	52	103
Carbon fixation in photosynthetic organisms	38	37
Flavonoid biosynthesis	87	33
Fatty acid elongation	8	22
Sulfur metabolism	28	13
Tropane, piperidine and pyridine alkaloid biosynthesis	13	24
Valine, leucine and isoleucine degradation	11	29
Isoquinoline alkaloid biosynthesis	12	12
Cutin, suberine and wax biosynthesis	29	46
Propanoate metabolism	19	20
