# Supplementary material for: Transcriptome profiling analysis reveals the role of silique in controlling seed oil content in Brassica napus
Source: PLoS One. 2017 Jun 8;12(6):e0179027. doi: 10.1371/journal.pone.0179027 (PMC5464616; doi:10.1371/journal.pone.0179027)
Supplement: S4 Table — (PDF) [file pone.0179027.s010.pdf]

S4 Table. Overview of the most enrichment of 30 different expression KEGG pathways in HFA15-vs-LFA15

Pathway	Up-regulated genes number	Down-regulated genes number
Valine, leucine and isoleucine degradation	25	27
Glutathione metabolism	43	56
Synthesis and degradation of ketone bodies	4	4
Arginine and proline metabolism	52	49
Ribosome	214	110
Linoleic acid metabolism	12	13
Tropane, piperidine and pyridine alkaloid biosynthesis	12	31
Vitamin B6 metabolism	8	6
alpha-Linolenic acid metabolism	30	45
Fatty acid metabolism	23	33
Nitrogen metabolism	22	36
Fatty acid elongation	15	17
Zeatin biosynthesis	29	34
Glycerophospholipid metabolism	47	70
Lysine degradation	18	18
Ether lipid metabolism	11	22
Peroxisome	34	56
Sphingolipid metabolism	18	26
Ascorbate and aldarate metabolism	25	40
Amino sugar and nucleotide sugar metabolism	57	72
Pentose phosphate pathway	26	26
Biotin metabolism	4	0
Nicotinate and nicotinamide metabolism	6	7
Glycerolipid metabolism	26	36
Lipoic acid metabolism	3	3
Propanoate metabolism	18	23
Phenylalanine, tyrosine and tryptophan biosynthesis	24	22
Indole alkaloid biosynthesis	16	9
Inositol phosphate metabolism	34	38
Monoterpenoid biosynthesis	6	3
