# Supplementary material for: Transcriptome profiling analysis reveals the role of silique in controlling seed oil content in Brassica napus
Source: PLoS One. 2017 Jun 8;12(6):e0179027. doi: 10.1371/journal.pone.0179027 (PMC5464616; doi:10.1371/journal.pone.0179027)
Supplement: S5 Table — (PDF) [file pone.0179027.s011.pdf]

S5 Table. Overview of the most enrichment of 30 different expression KEGG pathways in HFA25-vs-LFA25

Pathway	Up-regulated genes number	Down-regulated genes number
Glutathione metabolism	38	63
Sulfur metabolism	9	42
Phenylalanine, tyrosine and tryptophan biosynthesis	17	39
Fatty acid biosynthesis	32	15
Valine, leucine and isoleucine degradation	31	16
Propanoate metabolism	28	18
Sphingolipid metabolism	25	21
Amino sugar and nucleotide sugar metabolism	70	60
Valine, leucine and isoleucine biosynthesis	22	24
Phosphatidylinositol signaling system	42	49
alpha-Linolenic acid metabolism	38	34
Indole alkaloid biosynthesis	16	12
Glycerophospholipid metabolism	58	55
Pyruvate metabolism	50	42
Ascorbate and aldarate metabolism	33	31
Cysteine and methionine metabolism	33	58
Linoleic acid metabolism	12	9
Fatty acid elongation	16	14
Vitamin B6 metabolism	3	9
Nitrogen metabolism	27	27
Glyoxylate and dicarboxylate metabolism	29	40
Selenocompound metabolism	7	13
Pantothenate and CoA biosynthesis	15	17
Starch and sucrose metabolism	141	126
Inositol phosphate metabolism	39	32
Taurine and hypotaurine metabolism	12	6
Glycerolipid metabolism	28	31
C5-Branched dibasic acid metabolism	3	5
Folate biosynthesis	8	8
Ether lipid metabolism	13	16
